# Water, nitrogen, and phosphorus coupling improves gray jujube fruit quality and yield

**DOI:** 10.1515/biol-2022-0863

**Published:** 2024-04-26

**Authors:** Xudong Zhang, Jingjing Wang, Xinlu Bai, Shijie An, Qiangqing Zheng, Zhihui Tang, Jinhu Zhi

**Affiliations:** College of Agriculture, Tarim University, Aral, 843300, China; Research Center of Oasis Agricultural Resources and Environment in Southern Xinjiang, Tarim University, Aral, Xinjiang 843300, China; Research Institute of Garden, Xinjiang Academy of Agricultural Reclamation Sciences, Shihezi, Xinjiang, 832000, China; Research Institute of Mechanical Equipment, Xinjiang Academy of Agricultural Reclamation Sciences, Shihezi, Xinjiang, 832000, China

**Keywords:** gray jujube, water and fertilizer integration, fruit quality, yield, comprehensive evaluation, yield model, economic model

## Abstract

Irrigation and fertilization are indispensable links in the jujube planting industry in southern Xinjiang, China. Regulating the relationship between fertilization and irrigation can effectively reduce costs and improve economic efficiency. A 2-year water and fertilizer optimization coupling test was conducted to determine the optimal water and nutrient supply scheme. The three-factor randomized block experiment included water (W), nitrogen (N), and phosphorus (P). According to the principal component analysis of each index, each treatment’s comprehensive score was obtained. Using yield and economic regression models, the theoretical value and yield value of the optimal economic benefit are inferred. When W, N, and P were applied together, the fruit quality and yield of each treatment significantly differed, and the vitamin C, soluble sugar, and sugar-acid ratio increased significantly with an increase in N fertilizer. However, the titratable acid decreased. An increase in irrigation and nitrogen application significantly increased fruit yield. The comprehensive score was the highest in the N4P3W2 treatment, which improved fruit quality, and the lowest in the N3P3W2 treatment. When the amounts of N, P, and W were 275.56 kg hm^−2^, 413. 66 kg hm^−2^, and 7278.19 m^3^ hm^−2^, respectively, the theoretical economic benefit was the best. The N4P3W2 treatment is the optimal treatment.

## 1 Introduction

China has 98% of the worldwide jujube resources that are widely distributed in Heilongjiang, Jilin, Tibet, and Xinjiang [1]. Because of its rich nutritional and high economic value, jujube is the main economic crop planted in China. The development of jujube leaves is affected when the water supply is insufficient in the early stages of development [2]. Water and fertilizer integration is an agricultural technology combining irrigation and fertilization in which the fertilizer and water mixture is applied to the rhizosphere soil layer [3]. Compared with traditional agricultural irrigation technology, water and fertilizer integration technology can accurately control the amount of irrigation and fertilization and improve the utilization rate of water and fertilizer [4]. Water and fertilizer integration technology is quick, efficient, accurate, controllable, labor-saving, and non-destructive, and water and fertilizer are supplied simultaneously [5] while also increasing production [6].

Owing to the large demand for water and fertilizer in the production process of fruit trees, traditional agricultural irrigation methods can easily cause excessive water and fertilizer use, leading to a low utilization rate of water and fertilizer and causing environmental pollution [7]. The integration of water and fertilizer can effectively improve the current situation of insufficient water and fertilizer use and improve fruit tree growth and soil fertility [8]. An experimental study on integrating water and fertilizer in Red Fuji apples found that this method can effectively improve fruit hardness, soluble solids, and single-fruit weight [9]. Furthermore, compared with the control, the number of green leaves, leaf length, and plant height of pineapples cultivated using integrated water and fertilizer increased by 12.90, 12.10, and 8.77%, respectively. Soluble sugar and vitamin C in the fruit were significantly improved compared to those in the control [10]. Furthermore, integrated water and fertilizer management with no-tillage and grass planting improved avocado growth and the soil microenvironment of orchards, increased soil organic matter content, loosened the soil, and reduced soil moisture loss during high-temperature seasons [11].

However, few studies have been conducted on the effects of water and fertilizer integration on the growth and fruit quality of fruit-bearing jujube trees in orchards. Therefore, the aim of this study is to elucidate the effects of different amounts of water and fertilizer on the quality of jujube fruits to determine the optimal amount of water and fertilizer to ensure quality and reduce costs and to provide theoretical support for studying water and fertilizer integration for jujube trees.

## 2 Materials and methods

### 2.1 Test site characteristics

The experiment was conducted at the sixth new company of the ninth regiment of Aral City, the first division of the Xinjiang Production and Construction Corps (E longitude 81°6′, N latitude 40°34′, 1,022 m above sea level). The experimental area belongs to the Tarim Basin, and receives sufficient sunshine, an annual rainfall of 40–82 mm, and an evaporation of 1,876–2,558 mm. Seven-year-old jujube trees (gray jujube variety, sour jujube rootstock) were used. The plant row spacing was 1 m × 6 m, the size of the test site was 0.2 hm^2^, and the soil type was sandy loam. Table 1 lists the basic physical and chemical properties of the soil samples from the experimental area.

**Table 1:** Basic physical and chemical properties of soil samples from the jujube orchard

Determination standard	Soil depth (cm)		
	0–20	20–40	40–60
Volumetric weight (g cm^−3^)	1.32	1.40	1.47
Alkaline hydrolysis nitrogen (mg kg^−1^)	23.28	17.62	15.33
Rapidly available phosphorus (mg kg^−1^)	30.28	27.33	23.57
Electrical conductivity (μS cm^−1^)	109.87	106.38	90.26
pH	8.14	8.23	7.86

### 2.2 Test schemes

This experiment was conducted between 2021 and 2022. Experiments 1 and 2 were three-factor randomized block experiments using water (W), nitrogen (N), and phosphorus (P) as influencing factors, and each factor was set at five levels. A total of 15 treatments were tested and each treatment was repeated 3 times for a total of 45 plots; experiment 2 consisted of 8 treatments and each treatment was repeated 3 times for a total of 24 plots. There were five trees in each plot, and the area of each tree was 6 m^2^. The amounts of N and P were expressed as the amount of pure nutrient. The first levels of N, P, and W were 300.0 kg hm^−2^, 270.0 kg hm^−2^, and 4400.0 m^3^ hm^−2^; the second levels were 397.5 kg hm^−2^, 352.5 kg hm^−2^, and 5600.0 m^3^ hm^−2^; the third levels were 495.0, 435.0 kg hm^−2^, and 6800.0 m^3^ hm^−2^; the fourth levels were 592.5 kg hm^−2^, 517.5 kg hm^−2^, and 8000.0 m^3^ hm^−2^; and the fifth levels were 690.0 kg hm^−2^, 600.0 kg hm^−2^, and 9200.0 m^3^ hm^−2^, respectively. The treatment number and the amount of N, P, and W in each treatment are shown in Table 2.

**Table 2:** Amount of water and fertilizer in different treatments applied to gray jujube trees

Year	Treatment	Fertilizer (pure nutrient) dosage (kg hm^−2^)			Irrigation capacity (m^3^ hm^−2^)
		N	P_2_O_5_	K_2_O	
2021	N1P3W1	300.0	435.0	270.0	4400.0
	N2P3W1	397.5	435.0	270.0	4400.0
	N3P3W1	495.0	435.0	270.0	4400.0
	N4P3W1	592.5	435.0	270.0	4400.0
	N5P3W1	690.0	435.0	270.0	4400.0
	N3P1W1	495.0	270.0	270.0	4400.0
	N3P2W1	495.0	352.5	270.0	4400.0
	N3P3W1	495.0	435.0	270.0	4400.0
	N3P4W1	495.0	517.5	270.0	4400.0
	N3P5W1	495.0	600.0	270.0	4400.0
	N1P1W1	300.0	270.0	270.0	4400.0
	N1P1W2	300.0	270.0	270.0	5600.0
	N1P1W3	300.0	270.0	270.0	6800.0
	N1P1W4	300.0	270.0	270.0	8000.0
	N1P1W5	300.0	270.0	270.0	9200.0
2022	N3P3W2	495.0	435.0	270.0	5600.0
	N3P4W2	495.0	517.5	270.0	5600.0
	N4P3W2	592.5	435.0	270.0	5600.0
	N4P4W2	592.5	517.5	270.0	5600.0
	N3P3W3	495.0	435.0	270.0	6800.0
	N3P4W3	495.0	517.5	270.0	6800.0
	N4P3W3	592.5	435.0	270.0	6800.0
	N4P4W3	592.5	517.5	270.0	6800.0

N1: 300.0 kg hm^−2^, N2: 397.5 kg hm^−2^, N3: 495.0 kg hm^−2^, N4: 592.5 kg hm^−2^, N5: 690.0 kg hm^−2^; P1: 270.0 kg hm^−2^, P2: 352.5 kg hm^−2^, P3: 435.0 kg hm^2^, P4: 517.5 kg hm^−2^, P5: 600.0 kg hm^−2^; W1: 4400.0 m^3^ hm^−2^, W2: 5600.0 m^3^ hm^−2^, W3: 6800.0 m^3^ hm^−2^, W4: 8000.0 m^3^ hm^−2^, W5: 9200.0 m^3^ hm^−2^.

The fertilizer types were urea (N: 46%), diammonium phosphate (N-P_2_O_5_: 18-46-0), and potassium sulfate (K_2_O: 50%). The fertilizer was dissolved in a fertilization tank and dripped into the soil using water pressure to control the amount of irrigation in each plot.

The trees were fertilized five times throughout the growth period. A germination fertilizer was applied in early April and during the late May flowering period. A strong fruit fertilizer was applied from the end of June to the beginning of July and applied once during the fruit expansion period in mid–late July. The last fertilization was applied in early and middle August. The trees were irrigated eight times throughout the growing period. The first irrigation was in the middle of April for germination. The trees were then watered once at the beginning and in late May during flowering. The trees were watered once in mid-June during the flowering period, and once in early July during the fruit-promoting period. Trees were watered once during fruit expansion in mid-July, once in late July at the white ripe stage, and, finally, watered once in early August.

### 2.3 Test indicators and methods

Fruits with no pests or diseases and of similar sizes were randomly collected from each tree at the fruit maturity stage 2 m above the ground to determine soluble sugar, reducing vitamin C (Vc), titratable acid, and other indicators.

(1) Soluble sugar was measured using anthrone colorimetry.
(2) The molybdenum blue colorimetric method was used to determine the Vc content.
(3) The titratable acid content was determined using standard sodium hydroxide titration [12].
(4) The sugar-to-acid ratio was calculated as the soluble sugar-to-titratable acid ratio.
(5) The yield was calculated by dividing the location of each plot, and the jujube fruit in each plot area was weighed.

### 2.4 Data analysis

Excel and SPSS 26.0 were used to process the data and analyze the conclusion. The soluble sugar (*X*1), titratable acid (*X*2), Vc (*X*3), sugar-to-acid ratio (*X*4), and yield (*X*5) of jujube fruit were used as the evaluation indices. The eigenvectors and eigenvalues of each index were obtained using principal component analysis using the SPSS 26.0 data processing system.

## 3 Results

### 3.1 Different water and fertilizer treatments improve fruit quality and yield

By analyzing and comparing the fruit quality and yield of “Huizao” in 2021 and 2022, there were no significant differences in the vitamin C content and sugar-acid ratio while there were significant differences in the soluble sugar content, titratable acid content, and yield in the fruits of each treatment in 2021 (Table 3). In 2022, there were significant differences in the vitamin C content and sugar-acid ratio in the fruits, but the difference in yield changed from significant to insignificant.

**Table 3:** Changes in jujube fruit quality and yield under each treatment

Year	Treatment	Vitamin C (g kg^−1^)	Soluble sugar (g kg^−1^)	Titratable acid (g kg^−1^)	Sugar-to-acid ratio	Yield (kg hm^−2^)
2021	N1P3W1	2.99a	137.94ef	1.47bcde	107.36a	3280.00de
	N2P3W1	2.94a	159.40cde	1.44bcde	104.74a	4150.00d
	N3P3W1	2.71a	191.59ab	1.33de	121.03a	5970.00c
	N4P3W1	3.04a	178.48abc	1.54bcde	106.96a	8060.00a
	N5P3W1	3.01a	179.67abc	1.70abcd	132.54a	7690.00ab
	N3P1W1	2.60a	133.17f	2.00a	84.93a	2650.00e
	N3P2W1	2.61a	142.71ef	1.57bcde	101.60a	3540.00de
	N3P3W1	2.91a	152.25def	1.25e	107.98a	5670.00c
	N3P4W1	3.21a	178.48abc	1.38cde	86.93a	5740.00c
	N3P5W1	3.02a	159.40cde	1.52bcde	136.93a	5390.00c
	N1P1W1	2.54a	152.25def	1.54bcde	104.61a	2830.00e
	N1P1W2	2.67a	170.13bcd	1.74abc	107.56a	3920.00d
	N1P1W3	2.85a	197.56a	1.47bcde	113.81a	7520.00ab
	N1P1W4	3.14a	184.44ab	1.60bcde	103.14a	6980.00b
	N1P1W5	2.90a	182.05abc	1.82ab	118.41a	5220.00c
	N	ns	3.35	3.93	ns	59.69
	P	4.90	4.03	8.71	3.30	24.95
	W	ns	5.44	3.37	ns	57.88
2022	N3P3W2	2.93d	145.87c	1.80a	83.83b	8360.00a
	N3P4W2	2.98d	177.10abc	1.73a	111.86b	9020.00a
	N4P3W2	3.78a	205.47a	0.90b	224.42a	9570.00a
	N4P4W2	3.09d	194.27ab	1.47ab	140.02b	9350.00a
	N3P3W3	3.49abc	154.70c	1.73a	90.64b	9460.00a
	N3P4W3	3.19cd	165.80bc	1.83a	90.18b	9350.00a
	N4P3W3	3.62ab	210.17a	1.33ab	157.34b	9020.00a
	N4P4W3	3.31bcd	196.83ab	1.33ab	147.52b	9460.00a
	N × P	7.75	6.08	ns	6.31	ns
	N × W	6.57	ns	ns	ns	4.52
	P × W	ns	ns	ns	ns	ns
	N × P × W	7.06	ns	ns	4.52	ns

The letters in the table represent significant differences (*p* < 0.05). N1: 300.0 kg hm^−2^, N2: 397.5 kg hm^−2^, N3: 495.0 kg hm^−2^, N4: 592.5 kg hm^−2^, N5:690.0 kg hm^−2^; P1: 270.0 kg hm^−2^, P2: 352.5 kg hm^−2^, P3: 435.0 kg hm^−2^, P4: 517.5 kg hm^−2^, P5: 600.0 kg hm^−2^; W1: 4400.0 m^3^ hm^−2^, W2: 5600.0 m^3^ hm^−2^, W3: 6800.0 m^3^ hm^−2^, W4: 8000.0 m^3^ hm^−2^, W5: 9200.0 m^3^ hm^−2^.

The treatment with the highest soluble sugar content in the first year of fruiting was N1P1W1 (197.56 g kg^−1^), which was 48.4% higher than that of the lowest treatment N3P1W1. The treatment with the highest titratable acid content was N3P1W1 (2.00 g kg^−1^), which was 60.0% higher than that of the lowest treatment N3P3W1. The treatment with the highest yield was N4P3W1 (8,060 kg hm^−2^), which was 200.0% higher than that of the lowest treatment N3P1W1.

The treatment with the highest vitamin C content in the second year was N4P3W2 (3.75 g kg^−1^), which was 29.0% higher than that of the lowest treatment N3P3W2. The treatment with the highest soluble sugar content was N4P3W3 (210.17 g kg^−1^), which was 44.1% higher than that of the lowest treatment N3P3W2.The treatment with the highest titratable acid content was N3P4W3 (1.83 g kg^−1^), which was 100.0% higher than that of the lowest treatment N4P3W2. The treatment with the highest sugar-acid ratio was N4P3W2 (224.42); compared with the lowest treatment N3P3W2, it increased by 167.7%.

The results showed that increasing the amount of N, P, and W could increase the soluble sugar content and yield while reducing the titratable acid content, but excessive N, P, and W would reduce the soluble sugar content and yield while increasing the titratable acid content. Via the interactions of N, P, and W, the quality, content, and yield of fruits can be further improved.

### 3.2 Principal component analysis based on yield and fruit quality

The results of the principal component analysis are shown in Tables 4 and 5. The cumulative contribution rate of the first two principal component variances was 82.4160% (because the eigenvalues of the first two principal components were >1, the principal components *F*3–*F*5 include 17.5840% of the original data variation, and the principal component eigenvalues were <1; therefore, the data are not listed in Table 5), which contains most of the variation information in the original data. Therefore, the first two principal components were selected to evaluate the effects of irrigation and fertilization on the yield and fruit quality of the main fruit type. The variance contribution rate of the principal component *F*1 was 62.345%, and the load on the yield index was large. The principal component *F*1 could be defined as the 9 yield factor. The variance contribution rate of the principal component *F*2 was 20.071%, and the load on the quality index was large. The principal component *F*2 could be defined as the quality factor. According to Table 4, the equations can be established as follows:

(1) \[F=0.7560\times F1+0.2440\times F2,]\]

(2) \[F1=0.2461\times X1-0.2704\times X2+0.2666\times X3+0.1577\times X4+0.3019\times X5,]\]

(3) \[F2\text{}=\text{}0.2821\times X1+0.4236\times X2+0.0004\times X3+0.8133\times X4-0.2758\times X5.]\]

**Table 4:** Feature vectors of principal components affecting jujube yield and fruit quality

Principal component	Factor				
	*X*1	*X*2	*X*3	*X*4	*X*5
*F*1	0.2461	−0.2704	0.2666	0.1577	0.3019
*F*2	0.2821	0.4236	0.0004	0.8133	−0.2758

**Table 5:** Eigenvalue and variance contribution rate of the principal components affecting the yield and fruit quality of jujube trees

Principal component	Eigenvalue	Variance contribution rate (%)	Cumulative variance contribution rate (%)	Eigenvector (*λ*)
*F*1	3.1170	62.3450	62.3450	0.7560
*F*2	1.0040	20.0710	82.4160	0.2440

### 3.3 Comprehensive evaluation based on fruit yield and quality

The corresponding positions of leaf nutrients, yield, and fruit quality of jujube under different water and fertilizer treatments can be obtained using the factor scores of each principal component in Tables 4 and 5 to select the combination of water and fertilizer with a positive effect on the growth and development of jujube trees and fruit quality. Table 6 shows that the nutrient and quality factor scores of jujubes under different treatments varied. The order of comprehensive evaluation of the principal component analysis of each treatment was N4P3W2 > N4P3W3 > N4P4W3 > N4P4W2 > N3P3W3 > N3P4W3 > N3P4W2 > N3P3W2.

**Table 6:** Comprehensive evaluation of water and fertilizer treatment on yield and fruit quality of jujubes

Treatment	*F*1	*F*2	*F*	Comprehensive rank
N3P3W2	−1.308	−0.953	−1.221	8
N3P4W2	−0.579	−0.201	−0.487	7
N4P3W2	1.661	−0.205	1.206	1
N4P4W2	0.082	−0.053	0.049	4
N3P3W3	−0.427	0.958	−0.090	5
N3P4W3	−0.646	0.672	−0.325	6
N4P3W3	0.761	−0.307	0.501	2
N4P4W3	0.456	0.088	0.366	3

### 3.4 Establishment of the yield and economic models

#### 3.4.1 Establishment of the yield model

The regression model of yield (Table 3) with N, P, and W dosages was obtained using ternary quadratic regression fitting between yield and N, P, and W dosages in each experimental treatment. The significance test of equation (4) found that *R* ^2^ = 0.849, indicating that the degree of fit between the predicted and true values was good.

(4) \[Y=-27.6450\times \text{N}-10.7630\times \text{P}+2.8700\times \text{W}-0.0010\times \text{N}2-0.0630\times \text{P}2-0.0002\times \text{W}2+0.1160\times \text{N}\times \text{P}-0.0020\times \text{N}\times \text{W}+0.0040\times \text{P}\times \text{W}.]\]

The primary item coefficients were −27.6450, −10.7630, and 2.8700, respectively, indicating that the influence of each factor on yield was W > P > N. The interaction coefficients were 0.1160, −0.0020, and 0.0040, respectively, indicating that the interaction between each factor and yield was N × P > P × W > N × W.

**Figure 1:**
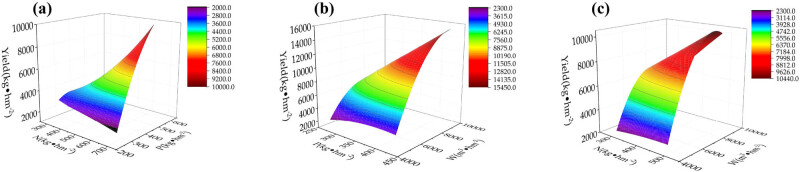
Interaction effects of each treatment on jujube fruit yield. W: water, P: phosphorus, and N: nitrogen. (a) Interaction effect of N and P on yield; (b) Interaction effect of P and W on yield; (c) Interaction effect of N and W on yield.

To explore the influence of coupling various factors on the yield, the factors in equation (4) were set at the lowest level and the following equations were obtained:

(5) \[Y=-36.4450\times \text{N}+6.8370\times \text{P}-0.0010\times \text{N}2-0.0630\times \text{P}2+0.1160\times \text{N}\times \text{P}+8756.0000,]\]

(6) \[Y=24.0370\times \text{P}+2.2700\times \text{W}-0.0630\times \text{P}2-0.0002\times \text{W}2+0.0040\times \text{P}\times \text{W}-8383.5000,]\]

(7) \[Y=3.6750\times \text{N}+3.9500\times \text{W}-0.0010\times \text{N}2-0.0002\times \text{W}2-0.0020\times \text{N}\times \text{W}-7498.7100.]\]

the fourth level (517.5 kg hm^−2^), the yield reached a maximum. At this time,

The response surface of yield to nitrogen and phosphorus interaction was a non-linear surface with a downward opening. When the nitrogen fertilizer was at the third level (495.0 kg hm^−2^) and the phosphorus fertilizer was at the fourth level (517.5 kg hm^−2^), the yield reached a maximum. At this time, the yield decreased with an increase in nitrogen fertilizer (Figure 1a). The response surface of the interaction effect of water and phosphorus on yield was a non-linear surface with a downward opening. When the irrigation amount reached the highest level (9,200 m^3^ hm^−2^), the yield reached a maximum value. At this time, the amount of phosphate fertilizer was low, and a continued increase in the amount of phosphate fertilizer led to a decrease in yield (Figure 1b). The response surface of yield to water and nitrogen interaction was anon-linear surface with a downward opening. When the nitrogen fertilizer was at the third level (495.0 kg hm^−2^), an increase in the irrigation amount increased the yield. When the irrigation amount was 9,200 m^3^ hm^−2^, the yield reached a maximum (Figure 1c).

#### 3.4.2 Establishment of the economic model

The economic benefit of each treatment was calculated using the yield, W, fertilizer, and labor costs of the experiment. The regression models of economic benefit (Eb), N (CN), P (CP), and W (CW) were obtained using ternary quadratic regression fitting of the economic benefits and N, P, and W costs:

(8) \[\text{Eb}=\text{}67.8750\times \text{CN}+252.8760\times \text{CP}+130.0890\times \text{CW}-0.0140\times \text{CN}2-0.1330\times {\text{CP}}^{2}-0.0080\times \text{CW}2+\text{}0.1520\times \text{CN}\times \text{CP}-0.0270\times \text{CN}\times \text{CW}-0.0070\times \text{CP}\times \text{CW}-501443.802.]\]

A significance test of equation (8) found that *R* ^2^ = 0.975, indicating that the degree of fit between the predicted and true values was good. The following three equations can be obtained from the partial differential derivation of equation (8):

(9) \[\text{dEb}/\text{dCN}=\text{}-0.0280\times \text{CN}+0.1520\times \text{CP}-0.0270\times \text{CW}+67.8750,]\]

(10) \[\text{dEb/dCP}=\text{}0.1520\times \text{CN}-0.2660\times \text{CP}-0.0070\times \text{CW}+252.8760,]\]

(11) \[\text{dEb/dCW}=\text{}-0.0270\times \text{CN}-0.0070\times \text{CP}-0.0160\times \text{CW}+130.0890.]\]

Let dEb/dCN = 0, dEb/dCP = 0, and dEb/dCW = 0; the optimal solution of equation (8) is CN = 247.15, CP = 889.26, and CW = 7278.19. According to the formula, the amount of nitrogen, phosphorus and irrigation can be obtained. The amount of nitrogen (pure nutrient), phosphorus (pure nutrient) and irrigation are respectively: 275.56 kg·hm^−2^, 413.66 kg hm^−2^, and 7278.19 m^3^ hm^−2^, respectively. Substituting these values in equation (4), it is concluded that the theoretical yield under this condition is 8622.00 kg hm^−2^.

## 4 Discussion

Nitrogen plays different roles throughout the crop growing period. During fruit growth, N affects the sugar content in the fruit by affecting the acid invertase, neutral invertase, sucrose synthase-cleavage, sucrose synthase-synthesis, and sucrose phosphate synthase content. Nitrogen also affects malic acid, citric acid, and pyruvate production in the tricarboxylic acid cycle and glycolysis, pentose phosphate pathway, and gluconeogenesis cycles to change the total acid content. Simultaneously, the sucrose content in fruit decreased under high-temperature stress (28–35°C) in another study; however, an increase in the N application rate offset the negative effect of high-temperature stress, and this phenomenon reduced hexose metabolism and led to hexose accumulation by inhibiting hexose kinase activity [13]. Our study found that, with an increase in N fertilizer, the content of soluble sugar in fruit increased, whereas an excessive increase in P fertilizer reduced the content of soluble sugar in fruit, similar to the conclusion of Zhou et al. [14]. Similarly, an increase in N fertilizer directly affected the total soluble solids content in grape juice [15]. The utilization rate of N fertilizer in apple trees decreased under the influence of distribution merchandising price promotion; however, the soluble sugar content in the fruit increased with an increase in the amount of N fertilizer within a certain range [16].

Furthermore, our study showed a significant difference in the titratable acid content of fruits in each treatment. The amount of N fertilizer was negatively correlated with the titratable acid content of the fruit. Increasing the amount of N fertilizer within a certain range reduced the titratable acid content in the fruit, similar to the findings of other studies [17,18,19]. In contrast, the amount of P fertilizer was positively correlated with the titratable acid content of the fruit, similar to the results of other studies [20]. In another study, the titratable acid content in red-fleshed navel orange pulp decreased significantly when the amount of P fertilizer was increased compared to the treatment without phosphate fertilizer; however, the decrease in titratable acid content decreased gradually with an increase in P fertilizer [21]. The change in the irrigation amount had little effect on the titratable acid content of the fruit.

In this study, increasing the amount of N fertilizer significantly increased the Vc content in jujube fruit, and the effect of increasing the irrigation amount on the Vc content was less than that of N fertilizer change on Vc content. When the amount of P fertilizer was increased, the Vc content in the fruit decreased significantly, similar to the conclusions of previous studies [22,23]. The Vc content of pepper fruit is typically used as an indicator for evaluating the fruit quality. Appropriate N fertilizer application increased the activity of dehydroascorbate reductase (DHAR) in green-ripe fruit and monodehydroascorbate reductase (MDHAR) in red-ripe fruit [24]. DHAR generally maintains the AsA (the main biologically active form of Vc) pool and its redox state. DHA-to-AsA recycling in the green and MDHA-to-AsA recycling in the red ripe period increases with the increase in the N fertilizer application. Therefore, increasing the amount of N fertilizer or reducing the amount of P fertilizer can increase the Vc content in different fruits [20,25,26,27,28].

Changes in W and fertilizer application rates significantly affected jujube fruit yield. When the amount of N fertilizer and irrigation increased, the jujube fruit yield increased significantly; however, the effect of the N fertilizer application rate on yield was greater than that of the irrigation amount, similar to the results of previous research [29]. In another study, an increase in N and P fertilizer significantly increased maize yield. This yield increase was attributed to increased yield components, such as grain number per spike, grain weight per spike, and 1,000-grain weight [30]. Another study on rice found that the yield also significantly increased when the amount of N fertilizer, N content in rice grains, and straw were increased [31]. The irrigation mode is also a factor affecting yield. Under low N and K conditions, the drip irrigation mode significantly increased the yield compared to that of the non-drip irrigation mode; however, when the N and potassium fertilizers were increased, the effect of the irrigation mode change on yield was weakened [32].

## 5 Conclusion

The increase in N fertilizer affected the nutritional quality of fruits, effectively increasing the content of soluble sugar, Vc, and the sugar-acid ratio in fruits and reducing the content of titratable acid, thus increasing the flavor and taste. Phosphorus fertilizer can also increase the content of soluble sugars and Vc in the fruit but is less effective than increasing the amount of N fertilizer. Simultaneously, an increase in P fertilizer also leads to an increase in the titratable acid content in the fruit and affects the taste of jujube fruit. An increase in irrigation slightly increased the soluble sugar and Vc content but significantly increased the titratable acid content in the fruit, which reduced its flavor and taste.

The comprehensive score of the N4P3W2 treatment was the highest, and that of the N3P3W2 treatment was the lowest. Therefore, coupling W, N, and P under water and fertilizer integration effectively improves the fruit quality of trunk fruit-shaped gray jujubes. The fruit quality and yield improvement effect of the N4P3W2 treatment was the most significant.

When the N, P, and W dosages are 275.56 kg hm^−2^, 413.66 kg hm^−2^, and 7278.19 m^3^ hm^−2^, respectively, the theoretical economic benefit is the best, and the theoretical yield is 8622.00 kg hm^−2^. In conclusion, the N4P3W2 treatment was the best.

In this experiment, although the effect of water, nitrogen, and phosphorus coupling on the growth of Huizao tree was explored, the amount of water, nitrogen, and phosphorus in each growth period was not determined within a reasonable threshold. According to the completed test, it is expected that the water and fertilizer required for each period can be quantified in the subsequent test.
